# Psychological distress links to magnetic resonance enterography abnormalities in Crohn’s disease

**DOI:** 10.1186/s13244-026-02273-w

**Published:** 2026-04-21

**Authors:** Ruonan Zhang, Yaoqi Ke, Huasong Cai, Yangdi Wang, Lili Huang, Xiaodi Shen, Qingzhu Zheng, Luyao Wu, Qiaochu Zhao, Weikai Zheng, Dailin Li, Ren Mao, Zhoulei Li, Shaochun Lin, Xuehua Li, Zhenpeng Peng

**Affiliations:** 1 Department of Radiology, The First Affiliated Hospital of Sun Yat-Sen University, 58 Zhongshan II Road, 510080 Guangzhou, People’s Republic of China; 2 Shantou University Medical College, 22 Xinling Road, 515041 Shantou, People’s Republic of China; 3 Department of Gastroenterology, The First Affiliated Hospital of Sun Yat-Sen University, 58 Zhongshan II Road, 510080 Guangzhou, People’s Republic of China

**Keywords:** Crohn’s disease, Magnetic resonance enterography, Psychological distress

## Abstract

**Objectives:**

Emerging evidence underscores psychological distress as a potential modifier of clinical outcomes in Crohn’s disease (CD), though its etiological basis remains undetermined. This study investigated associations between psychological distress and intestinal abnormalities identified by magnetic resonance enterography (MRE) through serum neurotransmitter analysis.

**Materials and methods:**

In this prospective study, 105 patients with CD and 46 healthy controls (HCs) completed the State-Trait Anxiety Inventory (including the STAI-Trait and State scores), the Beck Depression Inventory, and the Perceived Stress Scale to assess psychological distress. CD patients underwent MRE and serum neurotransmitter profiling. 79 patients received repeat MRE and psychological evaluations during follow-up. Statistical analyses included correlation analysis, multivariable logistic regression, and Cox proportional hazards modeling.

**Results:**

CD patients exhibited higher psychological distress scores vs. HCs (all *p* < 0.001). Anxiety severity (STAI-Trait score) correlated with CD bowel stricture, perienteric effusion, mural T2WI hyperintensity and hyperenhancement (|*r* | =0.454–0.606; all *p* < 0.05). Multivariable analysis showed that STAI-Trait score influenced the odds of perienteric effusion (OR = 1.124, 95% CI [1.007, 1.255], *p* = 0.036). Longitudinal follow-up demonstrated significantly higher incidence of new-onset perienteric effusion in patients with elevated baseline STAI-Trait scores compared to those with low scores (62.1% vs. 15.4%, *p* < 0.001), with STAI-Trait being the strongest predictor (HR = 6.986, 95% CI [2.274, 21.465], *p* < 0.001). Both STAI-Trait score and perienteric effusion showed consistent associations with serum tryptophan and histidine levels (|*r* | =0.203–0.255; all *p* < 0.05).

**Conclusion:**

CD patients’ psychological state is associated with intestinal morphological changes on MRE. The associations of serum tryptophan and histidine levels with both psychological distress and MRE features provide preliminary support for gut-brain interaction in CD.

**Critical relevance statement:**

This study links psychological distress to macro-morphological intestinal changes detectable by MRE, potentially associated with neurotransmitters like tryptophan, thereby advancing our understanding of the emotional factors associated with gut pathophysiology in CD and underscoring the importance of integrated psychological and inflammatory monitoring.

**Key Points:**

- Psychological distress correlates with clinical outcomes in patients with Crohn’s disease, but the reason for this association remains largely unexplored.
- Psychological distress is associated with macro-morphological changes as detected in MR enterography, blood tryptophan and histidine, and longitudinal follow-up further explains this connection.
- This study provides additional evidence for improving the management of both psychological well-being and intestinal inflammation in patients with Crohn’s disease.

**Graphical Abstract:**

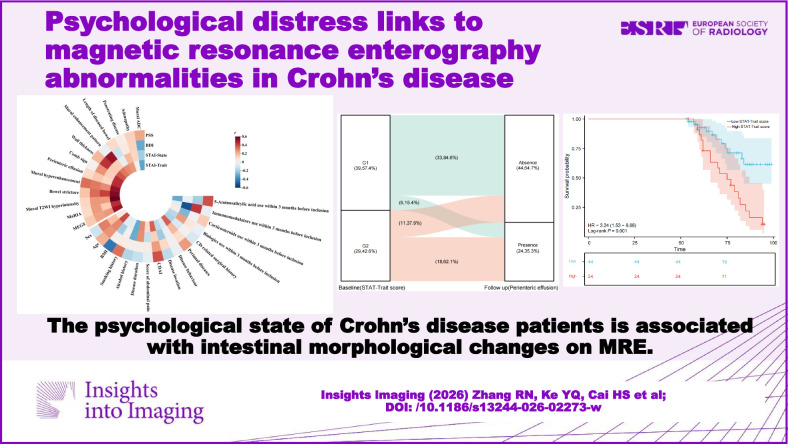

## Introduction

Psychosocial factors significantly impact the course of Crohn’s disease (CD) through the pivotal role of the brain-gut axis—a bidirectional communication network that links intestinal inflammation with the central nervous system [1, 2]. Within this network, psychological distress (encompassing anxiety, depression, and perceived stress) has emerged as a key modifier of clinical outcomes. Studies indicate that psychological distress is not only highly prevalent in CD [3] but is also associated with adverse clinical outcomes, including disease relapse and increased hospitalization [4, 5]. Furthermore, psychological interventions can modulate inflammatory biomarkers, providing compelling evidence for their clinical management relevance [6].

Despite this evidence, the interplay by which psychological distress influences the CD disease course remains unclear. We hypothesize that its effect is mediated by driving structural intestinal remodeling, manifesting as strictures, penetrating disease, or perienteric effusion—pathological features that are the principal causes of clinical disease progression in CD [7]. Magnetic resonance enterography (MRE) allows for the precise characterization of such transmural and extramural alterations [8, 9]. Therefore, investigating the link between psychological distress and MRE findings could offer direct insights into the brain-gut interaction.

To biochemically anchor this psychointestinal link, we turn to serum neurotransmitters. Specific neurotransmitters not only correlate with psychological states [10–12] but also directly disrupt intestinal barrier integrity, promoting inflammation [13]. Thus, they may serve as a plausible biochemical bridge. This study integrates psychological assessment, serum neurotransmitter profiling, and MRE to investigate the relationship between psychological distress and intestinal morphological changes in CD, aiming to provide a multidimensional understanding that could inform future psychological management strategies.

## Materials and methods

### Recruitment of study participants

The study was approved by the institutional ethics review board of our hospital (No. (2021)215-2). Informed consent was obtained from all participants. From June 2021 to May 2023, patients diagnosed with CD based on standard clinical, imaging, endoscopic, and histological criteria were prospectively recruited from our hospital.

The inclusion criteria were as follows: (a) adult patients with CD (≥ 18-year-old); (b) underwent MRE examination; (c) had blood samples available within one week of MRE; (d) simultaneously completed the State-Trait Anxiety Inventory (STAI) [14, 15], Perceived Stress Scale (PSS) [16, 17], and Beck Depression Inventory (BDI) [18, 19], to assess their psychological conditions; (e) patients included in the follow-up cohort were monitored for a minimum duration of six months and re-evaluated by MRE and psychological questionnaires. The exclusion criteria were as follows: motion or breathing artifacts, or metal implants that interfered with the MRE images.

We also recruited healthy participants as controls through community-wide open advertisements, matching their age and sex with patients with CD. HCs were eligible for participation if they met the following criteria: (1) absence of gastrointestinal symptoms or disease in the three months before inclusion; (2) no history of psychoneurological disorders or prior use of psychotropic medications; (3) completed the above psychological scale surveys.

**Fig. 1:**
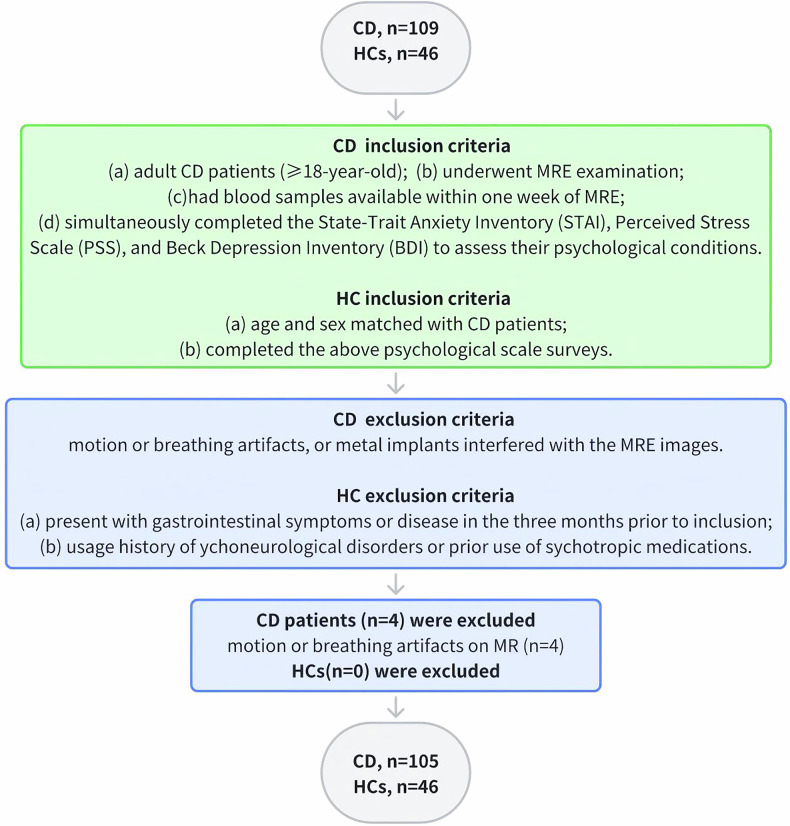
Flowchart of patient recruitment. CD, Crohn’s disease; HCs, healthy controls; MRE, magnetic resonance enterography

An a priori power analysis was conducted using G*Power to ensure an adequate sample size. The result determined that a minimum of 84 participants was needed to detect a medium correlation (|r | = 0.30) with 80% power at a 5% significance level (*α* = 0.05) between the primary psychological and MRE variables. Finally, a total of 105 patients were enrolled in our study. See Fig. 1 for a detailed description of the recruitment process.

### Collection of clinical characteristics of participants

Demographic data, including sex, age, body mass index (BMI), smoking, and alcohol history, were simultaneously collected from all participants when they completed psychological scale surveys. The clinical disease activity of patients with CD was evaluated by using CDAI [20, 21]. Montreal classifications [22, 23], disease duration, medication history (including current use of biologics, corticosteroids, immunomodulators, or 5-aminosalicylic acid therapy), and CD-related surgical history were noted from medical records. Moreover, we assessed the severity of daily abdominal pain in patients using the Visual Analogue Scale (VAS) [24, 25], which ranges from a score of 0 to 10. A score of 0 indicates the absence of pain, while a score of 10 represents excruciating pain.

### Psychological data assessment

A physician trained in psychological assessment accompanied the participants to assist them in completing the psychological scales. The physician provided guidance if the participants had any questions or difficulties understanding the items on the scales.

STAI is a self-report measure of the presence and severity of current symptoms of anxiety and generalized tendency to be anxious [15]. There are two subscales (including STAI-State score and STAI-Trait score) within this scale, each of which has 20 items (total score ranging from 20 to 80). The STAI-State score assesses the current state of anxiety. While the STAI-Trait score evaluates relatively stable aspects of “anxiety proneness”, including general states of calmness, confidence, and security. A cut-off score of 57 indicates clinically significant symptoms of anxiety for the STAI-State, while a threshold of 46 is used to detect a higher generalized tendency to be anxious for the STAI-Trait [14].

BDI is a 21-item self-report scale (total score ranging from 0 to 63), with a score of ≥ 14 indicating elevated symptoms of depression. Severity was categorized into mild (total BDI score 14–19), moderate (20–28), and severe (29–63) [18].

PSS is a 14-item scale (total score ranging from 0 to 56) that measures the extent to which respondents find their lives unpredictable, uncontrollable, and overwhelming. The total possible score on the PSS ranges from 0 to 56, with higher scores indicating elevated symptoms of stress. Severity was also categorized into mild (total PSS score 0–28), moderate (29–42), and severe (43–56) [17]. These psychological questionnaires are shown in the Supplementary Material 1. All patients included in the study underwent a comprehensive psychological assessment at baseline. Furthermore, those who proceeded to the follow-up phase were re-assessed during subsequent follow-up visits.

### Evaluation of intestinal alterations using MRE

The MRE protocol, including T2-weighted imaging (T2WI), fat suppression T2WI, diffusion-weighted MRI (DWI), and pre-/post-enhancement T1-weighted imaging, was performed for patients with CD with a 3.0-T MRI scanner (MAGNETOM Prisma; Siemens Healthineers; Supplementary Material 2).

**Fig. 2:**
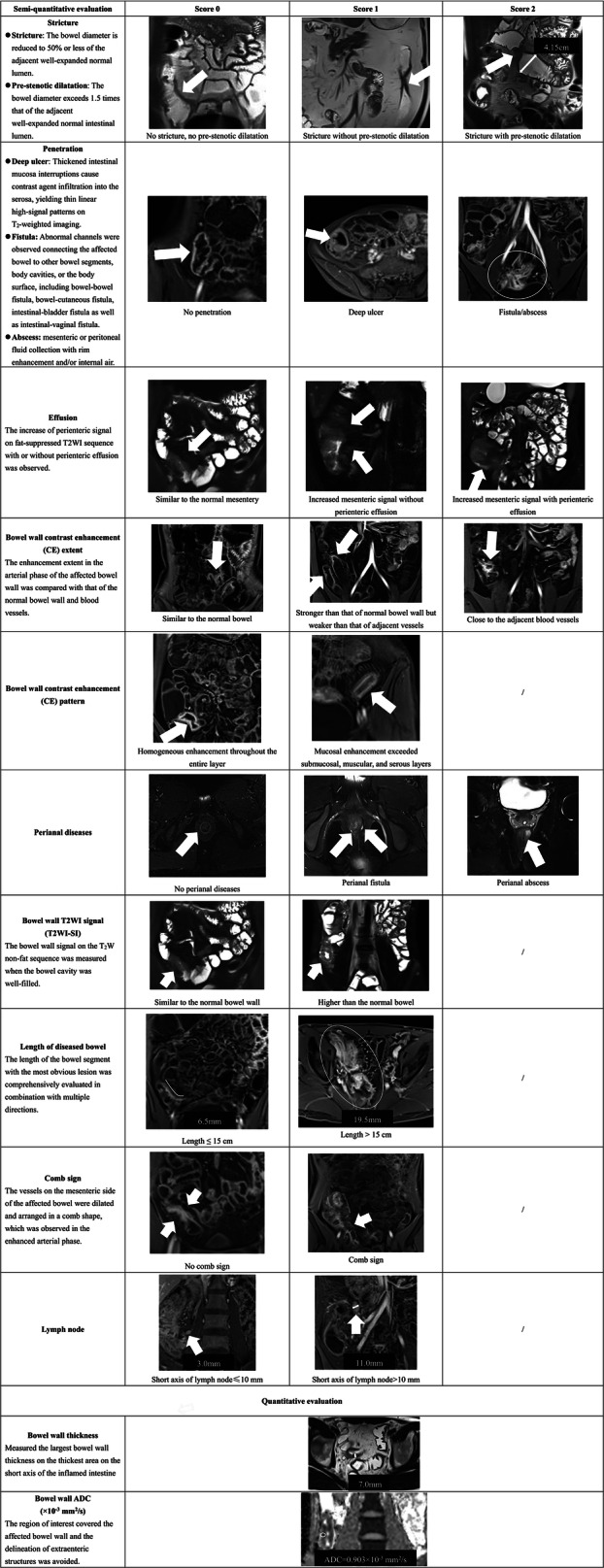
Definitions and illustrations of MRE features. MRE, magnetic resonance enterography; T2WI, T2-weighted imaging; ADC, apparent diffusion coefficient (all representative MRE images used in this figure were selected from the study cohort)

A total of twelve MRE features were assessed. MRE findings of the most severely diseased intestine for each patient were jointly evaluated and reached a consensus by three senior radiologists with more than 10 years of MRE diagnostic experience, who were blinded to patient’s clinical information and the results of psychological questionnaires (The detailed procedure for blinded evaluation is described in Supplementary Material 3). Among them, two MRE features, including wall thickness and mural apparent diffusion coefficient (ADC), were quantified and measured by putting two to three regions of interest (ROIs) on the target bowel wall. The average value of the measurements obtained from these ROIs was used for analysis. The other ten MRE features were assessed in a semi-quantitative manner, including bowel stricture, penetrating disease, perienteric effusion, comb sign, mural T2WI hyperintensity, mural hyperenhancement, mural enhancement pattern, adenopathy, length of diseased bowel, and perianal diseases. These semi-quantitative items were scored on a scale of 0–1 (0 indicating none and 1 indicating presence) or on a scale of 0–2 (0 indicating none, 1 indicating mild, and 2 indicating significant), based on their respective definitions [26] (Fig. 2). Subsequently, using the above MRE features, the Magnetic Resonance Index of Activity (MaRIA) [9, 27] and Magnetic Resonance Enterography Global Score (MEGS) [28, 29] were calculated to assess transmural inflammation (Supplementary Material 3). All patients included in this study underwent MRE examination at baseline. Furthermore, participants who progressed to the follow-up stage underwent repeat MRE examinations during subsequent follow-up visits.

### Blood neurotransmitter assessment

Blood samples were collected from patients with CD for targeted metabolomics profiling at baseline, with the specific aim of exploring the associations between psychological scores, neurotransmitter levels, and MRE features within this cohort. For comparative analysis, serum neurotransmitter levels were also measured in 30 age- and sex-matched healthy controls using the same targeted metabolomics protocol. Blood samples were drawn after overnight fasting, sent directly to the laboratory for serum collection, and frozen at −80 °C until analysis. Targeted metabolomics profiling methods used for detecting serum neurotransmitters were based on a previously published study [30]. 19 neurotransmitters (GABA, dopamine, serotonin, tryptophan, kynurenine, tyramine, norepinephrine, threonine, *L*-alanine, *L*-proline, taurine, histidine, glutamine, aspartic acid, glutamic acid, tyrosine, dopa, phenylalanine, and arginine) were tested. Please see Supplementary Material 4 for detailed methods on procedures.

### Correlation analysis of psychological abnormalities and MRE abnormalities with neurotransmitter-based explanations

This study examined the association between baseline psychological abnormalities and MRE features, with longitudinal validation and potential exploration through neurotransmitter analysis. We used Pearson or Spearman tests to test the correlations among psychological distress, MRE features, and neurotransmitter levels, and multivariable logistic regression to evaluate psychological-MRE associations. Kaplan-Meier analysis with log-rank testing was used for assessing psychological status-based survival differences. A two-step Cox regression approach (univariate screening at *p* < 0.10 followed by multivariate modeling) incorporating psychological, clinical, and demographic predictors was conducted.

### Statistical analysis

Demographic data were summarized using frequency analyses. Differences between patients with CD and HCs were assessed using *χ*² tests (categorical variables) or independent *t*-tests (continuous variables). Other statistical methods are described in their respective sections. Analyses were performed in SPSS 26.0 or R 4.2.2, with significance set at *p* < 0.05 (two-tailed), except for univariate Cox screening (*p* < 0.10).

## Results

### Study participants

We initially recruited 109 patients with CD, of which four were excluded due to unsatisfactory image quality (motion or breathing artifacts), resulting in a final inclusion of 105 patients (male/female = 90/15). Meanwhile, all 46 HCs were included without any exclusions (male/female = 39/7) (Table 1). All baseline data were checked for completeness, and no missing values were found. Therefore, the baseline analysis was performed on the full analysis set (CD, *n* = 105； HCs, *n* = 46). According to the CDAI assessment of patients with CD, 37 patients (35.24%) achieved remission, 28 (26.67%) exhibited mild disease activity, and 40 (38.09%) experienced moderate to severe disease activity. In comparison to HCs, patients with CD exhibited a significantly lower likelihood of alcohol consumption and a lower BMI (both *p* < 0.001). No significant differences were observed for other clinical characteristics. 26 patients were lost to follow-up. The collected follow-up cohort data, which included 79 patients (male/female = 69/10), were also summarized in Table 1.

**Table 1:** Characteristics of patients with CD and healthy controls

Characteristics	Patients with CD (*n* = 105)	Healthy controls (*n* = 46)	*p-*value^a^	CD follow-up cohort (*n* = 79^d^)	CD lost-to-follow-up cohort (*n *= 26^d^)	*p-*value^a^
Sex, *n* (%)			0.992			0.612
Male	90 (85.71%)	39 (84.78%)		69 (87.34%)	21 (80.77%)	
Female	15 (14.29%)	7 (15.22%)		10 (12.66%)	5 (19.23%)	
Age, years, mean ± SD	29.31 ± 7.14	29.30 ± 6.02	0.881	30.16 ± 7.21	26.73 ± 6.38	0.033
BMI, kg/m^2^, mean ± SD	20.17 ± 3.35	22.68 ± 2.24	< 0.001	20.23 ± 3.46	19.97 ± 3.04	0.736
Smoking history, *n* (%)	13 (12.38%)	7 (15.22%)	0.636	12 (15.19%)	1 (3.85%)	0.178
Alcohol history, *n* (%)	13 (12.38%)	22 (47.83%)	< 0.001	12 (15.19%)	1 (3.85%)	0.178
Disease duration, months, median (IQR)	60 (24–105)	N/A	N/A	60 (24–108)	41 (15–89)	0.546
Score of abdominal pain^b^, median (IQR)	3 (1–5)	N/A	N/A	3 (1–5)	2 (1–5)	0.950
CDAI, *n* (%)		N/A	N/A			0.333
Remission (< 150)	37 (35.24%)			29 (36.71%)	8 (30.77%)	
Mild disease (150–220)	28 (26.67%)			23 (29.11%)	5 (19.23%)	
Moderate disease (220–450)	40 (38.09%)			27 (34.18%)	13 (50.00%)	
Severe disease (> 450)	0			0	0	
Disease location, *n* (%)		N/A	N/A			0.418
Terminal ileum	16 (15.24%)			11 (13.92%)	5 (19.23%)	
Colonic	4 (3.81%)			2 (2.53%)	2 (7.69%)	
Ileocolic	82 (78.10%)			63 (79.75%)	19 (73.08%)	
Upper gastrointestinal tract	3 (2.85%)			3 (3.80%)	0	
Disease behavior, *n* (%)		N/A	N/A			0.851
Non-stricturing, non-penetrating	36 (34.29%)			26 (32.91%)	10 (38.46%)	
Stricturing	37 (35.24%)			28 (35.44%)	9 (34.62%)	
Penetrating	32 (30.48%)			25 (31.65%)	7 (26.92%)	
Perianal diseases, *n* (%)		N/A	N/A			0.473
None	34 (32.38%)			28 (35.44%)	6 (23.08%)	
Fistula	59 (56.19%)			42 (53.16%)	17 (65.38%)	
Abscess	12 (11.43%)			9 (11.40%)	3 (11.54%)	
Drug use^c^, *n* (%)		N/A	N/A			0.222
Biologics	42 (40.00%)			28 (35.44%)	14 (53.85%)	
Corticosteroids	7 (6.67%)			5 (6.33%)	2 (7.69%)	
Immunomodulators	28 (26.67%)			24 (30.38%)	4 (15.38%)	
5-Aminosalicylic acid	15 (14.29%)			13 (16.46%)	2 (7.69%)	

This table presents the demographic and clinical characteristics of patients with CD and healthy controls, and compares baseline profiles between the CD follow-up cohort and those lost to follow-up. Key findings show that no significant differences were observed in the key baseline variables between the CD follow-up cohort and the lost-to-follow-up cohort. Data are presented as mean ± SD for data that follow a normal distribution, or as median (IQR) for data that do not follow a normal distribution, and the counting data are presented as frequency or rate

*CD* Crohn’s disease, *BMI* body mass index, *CDAI* Crohn’s Disease Activity Index, *SD* standard deviation, *IQR* interquartile range, N/A not applicable

^a^ Independent samples t-test for continuous data and *χ*^2^ for categorical data

^b^ Visual Analogue Scale is used to assess the severity of abdominal pain in CD patients, ranging from score 0 to 10. A score of 0 indicates the absence of pain, while a score of 10 represents excruciating pain

^c^ Medicine use within 3 months before inclusion

^d^ Among 109 enrolled patients, 26 were lost to follow-up, leaving 79 patients in this follow-up section

### Compared to HCs, patients with CD exhibited a higher incidence of psychological distress and significantly lower serum levels of key neurotransmitters

Among all patients with CD, the prevalence of anxiety, depression, and stress was 35.24%, 31.43%, and 24.76% respectively (evaluated by questionnaire of STAI, BDI, and PSS, respectively). The results of all psychological scales of HCs were within the normal ranges. Therefore, the total incidence of psychological abnormalities in patients with CD was higher than that in HCs in our study ((CD, 46.67%) vs. (HCs, 0%), *p* < 0.001). For the four specific psychological scales, except for the STAI-State score (*p* = 0.312), the STAI-Trait, PSS and BDI scores of patients with CD were significantly higher than those of the HCs (Table 2; all *p* < 0.001).

**Table 2:** Psychological characteristics of patients with CD and healthy controls

Psychological scales	Patients with CD (*n* = 105)	Healthy controls (*n* = 46)	*p*-value^a^
STAI, *n* (%)			
State anxiety	5 (4.76%)	0	0.312
Trait anxiety	37 (35.24%)	0	< 0.001
BDI, *n* (%)			< 0.001
No depression	72 (68.57%)	46 (100%)	
Mild depression	16 (15.24%)	0	
Moderate depression	11 (10.48%)	0	
Severe depression	6 (5.71%)	0	
PSS, *n* (%)			< 0.001
Mild stress	79 (75.24%)	46 (100%)	
Moderate stress	24 (22.86%)	0	
Severe stress	2 (1.90%)	0	

This table compares psychological profiles between patients with CD and HCs using standardized scales. Key findings show that CD patients had a significantly higher burden of trait anxiety, depression, and moderate-to-severe stress compared to HCs, whereas the prevalence of state anxiety did not differ significantly between groups. Data are presented as numbers (column %); group comparisons were performed using the *χ*² test.

*CD* Crohn’s disease, *STAI* State-Trait Anxiety Inventory, *BDI* Beck Depression Inventory, *PSS* Perceived Stress Scale, *SD* standard deviation, *N/A* not applicable

Serum levels of neurotransmitters were also compared between the groups. As shown in Table 3, the majority of neurotransmitters exhibited significant differences between CD patients and HCs. Specifically, serum levels of tryptophan, histidine, and GABA were consistently lower in CD patients compared to HCs (all *p* < 0.05).

**Table 3:** Serum levels of neurotransmitters in patients with CD and healthy controls

Neurotransmitters (μmol/L)	Patients with CD (*n* = 105)	Healthy controls (*n* = 30)	*p*-value^a^
GABA	0.146 ± 0.070	0.177 ± 0.033	0.020
Dopamine	0.013 ± 0.004	0.017 ± 0.004	< 0.001
Serotonin	1.572 ± 0.822	1.653 ± 0.735	0.626
Tryptophan	62.897 ± 25.478	65.915 ± 12.260	0.532
Kynurenine	3.100 ± 1.349	2.365 ± 0.642	0.005
Tyramine	0.032 ± 0.003	0.033 ± 0.001	0.182
Norepinephrine	0.075 ± 0.039	0.102 ± 0.028	0.001
Threonine	132.773 ± 99.598	82.219 ± 22.021	0.007
Alanine	299.577 ± 107.460	399.818 ± 78.647	< 0.001
Proline	179.502 ± 70.699	190.147 ± 57.340	0.451
Taurine	129.966 ± 61.953	94.551 ± 36.634	0.003
Histidine	99.739 ± 23.385	116.983 ± 18.666	< 0.001
Glutamine	619.756 ± 155.150	646.831 ± 136.037	0.389
Aspartic acid	25.055 ± 9.818	19.376 ± 7.569	0.004
Glutamic acid	53.516 ± 28.040	42.150 ± 20.306	0.041
Tyrosine	61.013 ± 14.979	69.810 ± 21.375	0.012
Dopa	0.025 ± 0.008	0.025 ± 0.005	0.654
Phenylalanine	70.837 ± 20.355	68.228 ± 16.182	0.520
Arginine	134.571 ± 21.941	157.936 ± 22.547	< 0.001

This table presents a comparison of serum neurotransmitter concentrations between patients with CD and healthy controls. Key findings show that CD patients exhibit significantly lower levels of GABA, tryptophan, and histidine than healthy controls. Data are presented as mean ± SD; group comparisons are performed using independent samples *t*-tests

*CD* Crohn’s disease, *GABA* gamma-aminobutyric acid, *SD* standard deviation

^a^ Independent samples t-test for continuous data

### Correlation of psychological scores with clinical factors in patients with CD

**Fig. 3:**
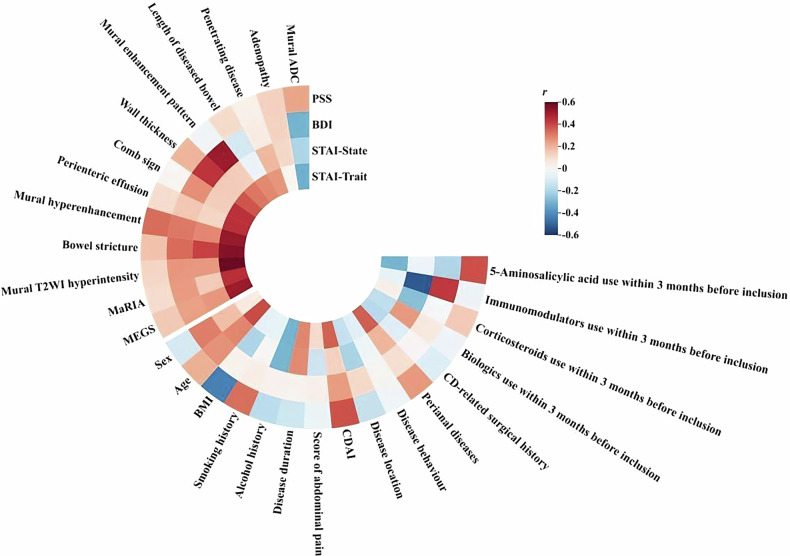
Correlation of psychological scores with clinical factors, MRE features, and inflammation scores in patients with CD. The correlation of psychological scores with clinical factors, MRE features and inflammation scores in patients with CD. BMI, body mass index; CDAI, Crohn’s Disease Activity Index; STAI, State-Trait Anxiety Inventory; BDI, Beck Depression Inventory; PSS, Perceived Stress Scale. MaRIA, Magnetic Resonance Index of Activity; MEGS, Magnetic Resonance Enterography Global Score; T2WI, T2-weighted imaging; ADC, apparent diffusion coefficient; STAI, State-Trait Anxiety Inventory; BDI, Beck Depression Inventory; PSS, Perceived Stress Scale. Red represents positive correlation, blue represents negative correlation, and the depth of color indicates the strength of correlation

The STAI-Trait score was positively associated with CDAI (*r* = 0.358, *p* < 0.05), perianal diseases (*r* = 0.359, *p* < 0.05), and age (*r* = 0.395, *p* < 0.05). Moreover, there was a positive correlation observed in patients with CD between the use of immunomodulator therapy within 3 months before inclusion and BDI scores (*r* = 0.431, *p* < 0.05), suggesting that patients with CD with a history of using this medication had higher BDI scores compared to those without. Besides, a positive correlation of PSS score with CDAI was observed (*r* = 0.385, *p* = 0.052), while it had a significantly negative correlation with BMI (*r* = –0.434, *p* < 0.05). No correlations were identified between STAI-State score and clinical factors in patients with CD (Fig. 3).

### Correlation of psychological scores with MRE features and inflammation scores in patients with CD

Due to the significant correlations between CDAI (reflecting clinical disease activity) and both STAI-Trait score and PSS score, we further investigated their correlation with MRE features (serving as objective indicators of intestinal morphology alterations and inflammation activity). After controlling for the covariates of sex, age, BMI, smoking history, alcohol history, CDAI, and perianal diseases, STAI-Trait score was significantly correlated with bowel stricture (*r* = 0.554), perienteric effusion (*r* = 0.454), mural T2WI hyperintensity (*r* = 0.606), mural hyperenhancement (*r* = 0.525), comb sign (*r* = 0.451), and bowel wall thickness (*r* = 0.380) (all *p* < 0.05) (Fig. 3). Furthermore, after controlling for the same covariates, STAI-Trait score was also correlated with MaRIA score (*r* = 0.453), and MEGS score (*r* = 0.517) (both *p* < 0.05) (Fig. 3). However, there were no statistically significant correlations found in the analysis between PSS score and MRE features as well as the two MRE inflammatory scores (all *p* > 0.05).

### Psychological distress, as reflected by STAI-Trait score, was a risk factor for intestinal abnormalities identified on MRE in patients with CD

**Fig. 4:**
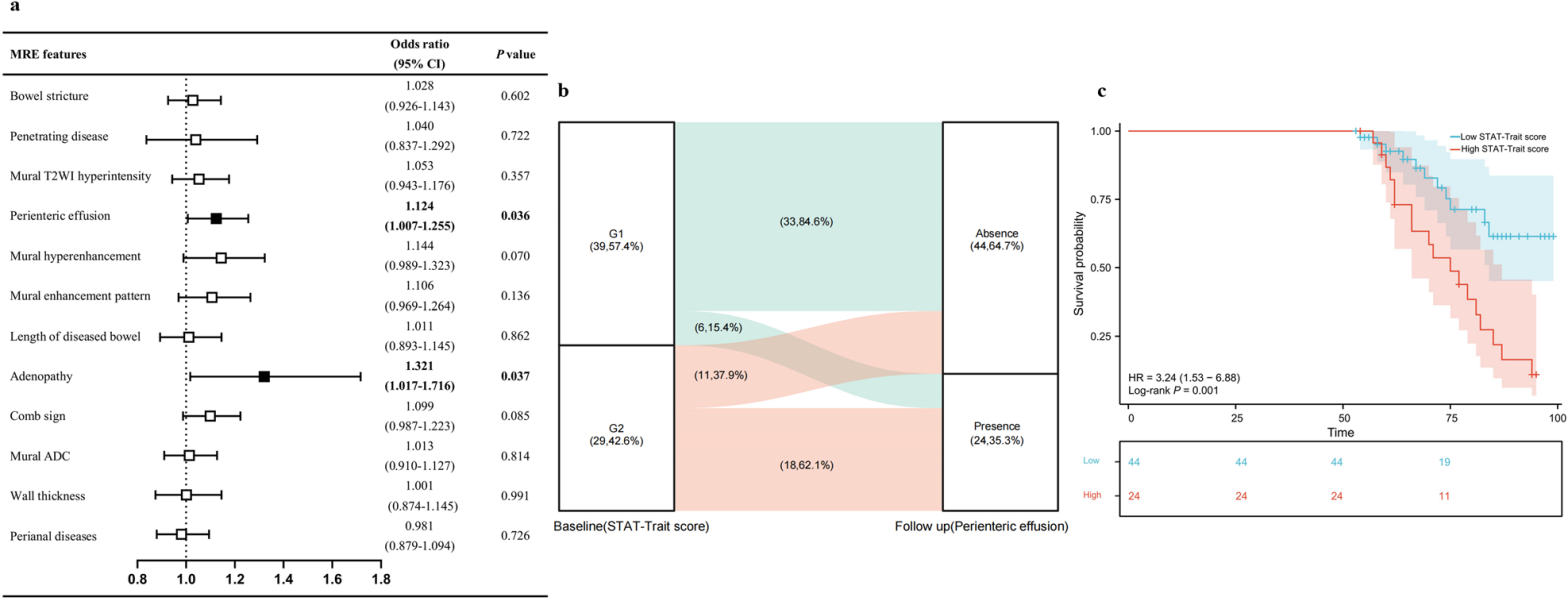
Psychological distress, as reflected by STAI-Trait score, was a risk factor for intestinal abnormalities identified on MRE in patients with CD. **a** Multivariable logistic regression analysis investigates the relationship between the psychological distress measured by STAI-Trait score and MRE features. Forest plot shows that the STAI-Trait score significantly influences the odds of perienteric effusion and adenopathy identified on MRE among patients with CD. MRE, magnetic resonance enterography; CI, confidence intervals; T2WI, T2-weighted imaging. **b** The incidence of perienteric effusion during follow-up varies among patients with different baseline psychological distress, as reflected by STAI-Trait score. A higher proportion of individuals with high STAT-Trait score at baseline experienced perienteric effusion during follow-up than those with low STAT-Trait score. The red arcs indicate the flow direction of patients with a baseline STAI-Trait score above the threshold score “46,” whereas the green arcs indicate the flow direction of patients with a baseline STAI-Trait score below the threshold. The width of the arcs corresponds to the proportion of patients in each category. The figure indicates the proportions of each component. G1, low STAT-Trait score at baseline; G2, high STAT-Trait score at baseline. **c** Kaplan–Meier survival curve shows that the median perienteric-effusion-free survival of patients with the high STAI-Trait score is significantly shorter than that of patients with a low STAI-Trait score (log-rank *p* = 0.001)

In light of the above findings, we further investigated the relationship between the STAI-Trait score that reflects psychological distress and a series of MRE features using multivariable logistic regression analysis. Our results indicated that the STAI-Trait score significantly influenced the odds of perienteric effusion identified on MRE among patients with CD (OR: 1.124, 95% CI [1.007, 1.255], *p* = 0.036), as well as adenopathy (OR: 1.321, 95% CI [1.017, 1.716], *p* = 0.037) (Fig. 4a). We further examined these relationships in the context of disease activity. We observed that patients with active CD (CDAI ≥ 150, *n* = 68) exhibited significantly lower serum levels of tryptophan, histidine, and GABA compared to those with remissive CD (CDAI < 150, *n* = 37) (all *p* < 0.05). Correlation analyses further demonstrated that CDAI scores were negatively correlated with tryptophan (*r* = −0.296), histidine (*r* = −0.454), and GABA (*r* = −0.334) (all *p* < 0.05). Stratified multivariate logistic regression analysis revealed that in the active disease group, STAI-Trait score remained a significant factor influencing the odds of perienteric effusion (OR: 1.154, 95% CI [1.004, 1.325], *p* = 0.043), whereas this association was not statistically significant in the remissive group ((OR: 1.093, 95% CI [0.841, 1.421], *p* = 0.505).

Building upon the association identified in our cross-sectional study, we conducted a longitudinal investigation to assess the relationship between STAI-Trait scores and perienteric effusion (an imaging marker more specific for intestinal inflammation than adenopathy [31, 32]). From the initial cohort of 105 enrolled patients, 79 patients adhered to scheduled follow-up visits. After excluding 11 patients with baseline perienteric effusion, we analyzed the remaining 68 patients by tracking their perienteric effusion status over a median follow-up period of 73 weeks. Participants were stratified by baseline STAI-Trait scores into: Group 1: Low STAI-Trait scores (*n* = 39); Group 2: High STAI-Trait scores (*n *= 29). The longitudinal analysis revealed significantly higher incidence of perienteric effusion in Group 2 (62.1% vs. 15.4%, *p* < 0.001; Fig. 4b). Kaplan-Meier analysis confirmed that patients with high baseline STAI-Trait scores had markedly shorter effusion-free survival (log-rank *p* = 0.001; Fig. 4c).

To evaluate the independent predictive value of STAI-Trait scores, we performed multivariate Cox regression incorporating variables selected through univariate screening: psychological scale scores, medication history, perianal disease, surgical history, CDAI, gender, age, and BMI (Table 4). The analysis identified STAI-Trait score as the sole significant independent predictor of perienteric effusion (HR = 6.986, 95% CI [2.274, 21.465], *p* < 0.001), while other variables showed no significant association (all *p* > 0.05).

**Table 4:** Univariate and multivariate Cox regression analysis of baseline factors for predicting perienteric effusion development in patients with CD

Characteristics	Total cohort (*n* = 68)	
	Hazard ratio (95% CI)	*p*-value
Univariate Cox regression analysis		
Psychological scales		
STAI-State score	1.019 (0.979–1.062)	0.352
STAI-Trait score	7.950 (2.806–22.520)	< 0.001
BDI	1.016 (0.982–1.051)	0.358
PSS	0.999 (0.948–1.052)	0.959
Clinical factors		
Age	0.999 (0.944–1.058)	0.983
Sex	0.765 (0.225–2.599)	0.668
BMI	0.962 (0.864–1.071)	0.476
CDAI	1.406 (0.622–3.179)	0.412
Perianal disease	0.393 (0.163–0.951)	0.038
Drug use^a^		
Biologic	0.978 (0.357–2.678)	0.966
Steroid/hormone	0.816 (0.304–2.190)	0.686
Immunomodulator	0.922 (0.374–2.273)	0.861
5-aminosalicylic acid	0.253 (0.059–1.078)	0.063
Surgical history	1.105 (0.489–2.497)	0.809
Multivariate Cox regression analysis		
STAI-Trait score	6.986 (2.274–21.465)	< 0.001
Perianal disease	1.021 (0.369–2.827)	0.968
5-aminosalicylic acid use during follow-up	0.359 (0.076–1.688)	0.195

This table presents Cox regression analyses of baseline factors associated with perienteric effusion development, with statistical significance defined as *p* < 0.100 for univariate and *p* < 0.050 for multivariate analysis. Key results demonstrate that a higher STAI-Trait score was a consistent and independent predictor of increased risk in both univariate (HR: 7.950, 95% CI [2.806, 22.520], *p* < 0.001) and multivariate analyses (HR: 6.986, 95% CI [2.274, 21.465], *p* < 0.001). Perianal disease and 5-aminosalicylic acid use were significant only in the univariate analysis

*STAI* State-Trait Anxiety Inventory, *BDI* Beck Depression Inventory, *PSS* Perceived Stress Scale, *BMI* body mass index, *CDAI* Crohn’s Disease Activity Index

^a^ Medicine use during follow-up

Overall, both cross-sectional and longitudinal follow-up studies have demonstrated a significant association between STAI-Trait score and perienteric effusion.

### Serum levels of tryptophan and histidine are significantly associated with psychological distress (reflected by STAI-Trait score) and intestinal abnormalities

**Fig. 5:**
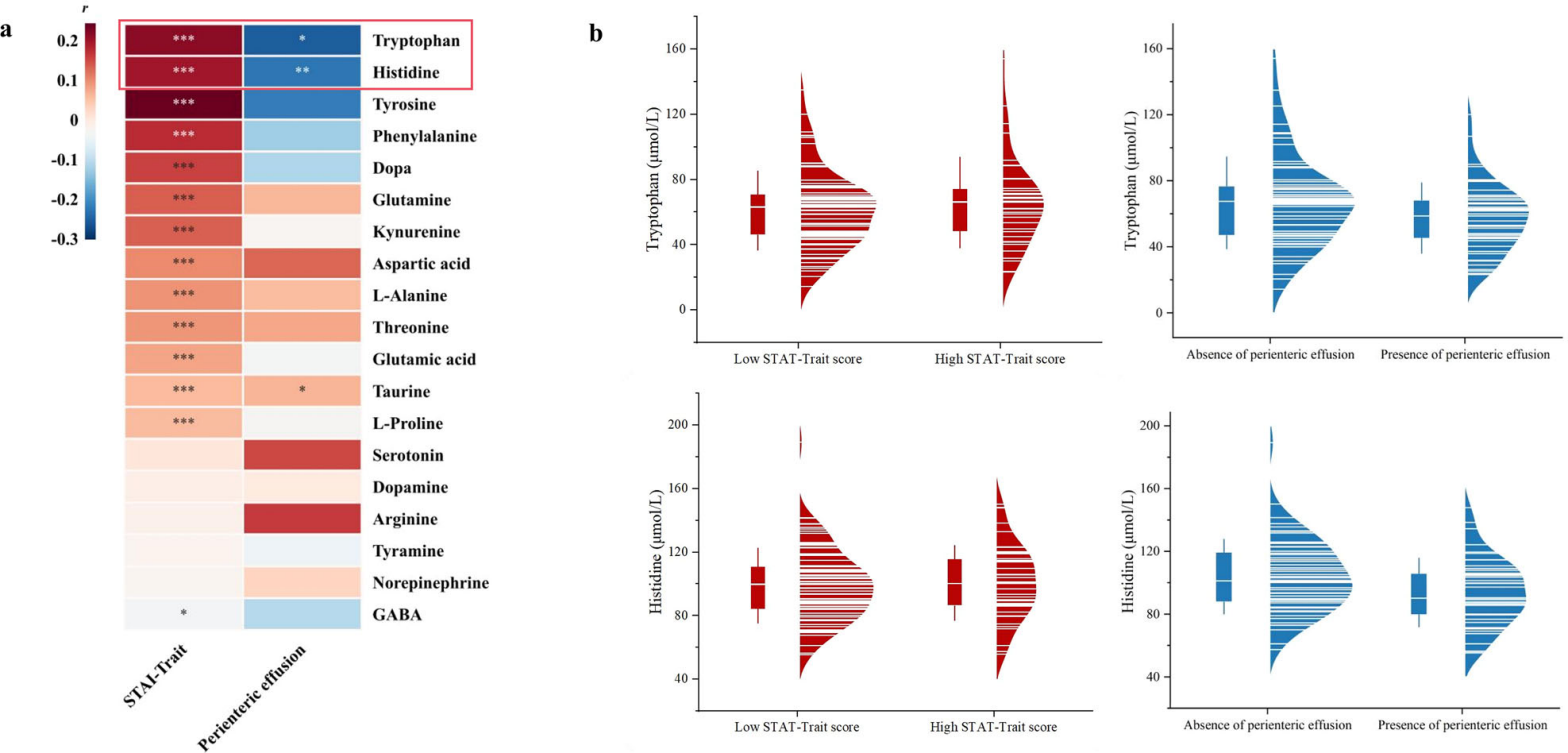
Serum neurotransmitter levels were associated with both psychological distress (STAI-Trait scores) and intestinal abnormalities. **a** The correlation of serum neurotransmitter levels with the STAI-Trait score and perienteric effusion in patients with CD. Both STAI-Trait score and perienteric effusion exhibit significant correlations with the serum levels of tryptophan and histidine. *significant with *p*-value < 0.05. **significant with *p*-value < 0.01. ***significant with *p*-value < 0.001. STAI, State-Trait Anxiety Inventory; GABA, Gamma-aminobutyric acid. Red represents positive correlation, blue represents negative correlation, and the depth of color indicates the strength of correlation. **b** Half-violin plots demonstrate the distribution of serum tryptophan and histidine levels in patients stratified by psychological status (with/without abnormalities) and perienteric effusion presence. STAI, State-Trait Anxiety Inventory

To further elucidate the link between psychological distress and intestinal abnormalities, we analyzed serum neurotransmitter levels, which are well-established biomarkers of emotional states [10–12]. After adjusting for sex, age, BMI, smoking history, and alcohol use, we found that higher STAI-Trait scores were significantly correlated with elevated serum levels of tryptophan (positive correlation, *r* = 0.220, *p* < 0.001), histidine (positive correlation, *r* = 0.203, *p* < 0.001), and tyrosine (positive correlation, *r* = 0.245, *p* < 0.001) (Fig. 5a, b). Conversely, perienteric effusion showed negative correlations with tryptophan (*r* = −0.220, *p* < 0.05) and histidine (*r* = −0.255, *p* < 0.05), but a positive correlation with taurine (*r* = 0.227, *p* < 0.05) (Fig. 5a, b). The bidirectional associations of tryptophan and histidine with both psychological distress and intestinal inflammation markers provided biochemical evidence to support the connection between psychological states and intestinal remodeling detected on MRE.

## Discussion

Our study demonstrates a significant and independent association between psychological distress and objective intestinal morphological alterations in CD, as assessed by MRE. This finding provides objective imaging evidence for the role of the brain-gut axis in CD, directly linking psychological factors to macro-morphological intestinal pathology.

This connection finds support in recent mechanistic explorations, where core pathways involving the brain-gut axis, neuroimmune interactions, and gut microbiota have been implicated [33]. Specifically, psychological stress is known to compromise intestinal barrier function, while chronic intestinal inflammation can activate neuroinflammatory pathways that contribute to anxiety and depressive behaviors [34]. Concurrently, the tryptophan metabolite pathway, serving as the precursor for serotonin (5-HT), plays a significant role in the pathogenesis of anxiety and depression [35] in CD patients. Extending this established framework, our investigation identified significant correlations between serum tryptophan and histidine levels with both psychological distress (STAI-Trait scores) and perienteric effusion. These findings not only provide supportive clinical cohort data for the hypothesized pathway of “neuroimmune dysregulation and impaired intestinal barrier integrity” but also offer visual, macro-morphological evidence through MRE for mechanisms previously described primarily at the molecular level.

Extending this line of evidence, we further observed that serum neurotransmitter profiles varied with both disease status and activity. Compared to HCs, CD patients showed consistently lower levels of tryptophan, histidine, and GABA. Moreover, among CD patients, those with active disease (CDAI ≥ 150) exhibited further reductions in these neurotransmitters compared to those in remission, and CDAI scores were inversely correlated with their concentrations. Critically, stratified analyses demonstrated that the association between psychological distress (STAI-Trait scores) and perienteric effusion remained significant even within the active disease subgroup, indicating that this link is independent of concurrent clinical disease activity. These findings collectively suggest that while neurotransmitter alterations are closely tied to CD presence and activity, the relationship between psychological factors and perienteric effusion represents a distinct and consistent pathway.

Regarding clinical implications, the intestinal morphological changes identified in our study likely represent an intermediate link between psychological distress and adverse clinical outcomes. Previous research has consistently linked psychological factors to hard clinical endpoints [36–41]; for instance, baseline depressive symptoms have been shown to predict subsequent increases in disease activity and higher hospitalization risk in CD patients. Our study advances this understanding by demonstrating that psychological distress (e.g., anxiety traits) is independently associated with objective intestinal inflammatory markers on MRE, such as perienteric effusion. This suggests that a key pathway through which psychological distress leads to adverse outcomes (e.g., disease relapse, hospitalization) may be the induction or exacerbation of objective intestinal morphological deterioration, thereby more tightly connecting the triad of “psychological state—objective intestinal inflammation—hard clinical endpoints” [42].

Methodologically, our use of MRE for objective assessment represents an advance over traditional reliance on clinical symptom indices. It is well recognized that the CDAI, commonly used in clinical practice, is influenced by subjective factors and may not fully accurately reflect the true state of intestinal inflammation. In recent years, the value of imaging assessment has become increasingly prominent. Whether through comprehensive prediction models built using ultrasonography or the advantages of MRE [9] (namely, lack of radiation and high soft-tissue resolution), these modalities provide more direct and objective information on intestinal pathology. Consequently, by linking psychological distress to robust objective MRE imaging features, our conclusions are more compelling than those relying solely on clinical indices or single biomarkers, thereby deepening the understanding of brain-gut interactions.

This study has several limitations that should be considered. First, the single-center design and male predominance in our cohort may limit the generalizability of our findings, warranting future multi-center studies with balanced gender representation. Second, the assessment of psychological distress relied on self-reported questionnaires, which are susceptible to subjective bias; incorporating structured clinical interviews or objective biomarkers in future research would strengthen the robustness of psychological data. Third, while revealing significant associations, our study design cannot delineate the directionality of brain-gut interactions. A deeper understanding of the underlying mechanisms requires future longitudinal studies. Finally, although we controlled for key confounders, the potential influence of unmeasured lifestyle factors (e.g., diet and sleep patterns) cannot be excluded. Subsequent investigations would benefit from employing integrated multi-omics approaches and systematic collection of lifestyle variables to provide a more comprehensive systems-level understanding.

In summary, our study integrates psychological assessment with MRE to demonstrate an independent association between psychological distress and objective intestinal changes in CD, providing imaging evidence for its link to adverse clinical outcomes. These findings support the clinical relevance of addressing psychological well-being in comprehensive CD management and highlight the need for future research to explore the brain-gut axis through longitudinal and interventional studies.

## Supplementary information

ELECTRONIC SUPPLEMENTARY MATERIAL

## Data Availability

All data, analytic methods, and study materials are available from the corresponding author (Zhenpeng Peng) upon reasonable request.

## Abbreviations

BDI: Beck Depression Inventory
BMI: Body mass index
CD: Crohn’s disease
CDAI: Crohn’s Disease Activity Index
CIs: Confidence intervals
GABA: Gamma-aminobutyric acid
HCs: Healthy controllers
HR: Hazard ratio
MaRIA: Magnetic Resonance Index of Activity
MEGS: Magnetic Resonance Enterography Global Score
MRE: Magnetic resonance enterography
OR: Odds ratio
PSS: Perceived Stress Scale
ROIs: Regions of interest
STAI: State-Trait Anxiety Inventory
T2WI: T2-weighted imaging
