# Supplementary material for: Psychological distress links to magnetic resonance enterography abnormalities in Crohn’s disease
Source: Insights Imaging. 2026 Apr 21;17:110. doi: 10.1186/s13244-026-02273-w (PMC13100207; doi:10.1186/s13244-026-02273-w)
Supplement: Supplementary file 1 — ELECTRONIC SUPPLEMENTARY MATERIAL [file 13244_2026_2273_MOESM1_ESM.pdf]

Psychological Distress Links to Magnetic Resonance Enterography Abnormalities in Crohn's Disease

ELECTRONIC SUPPLEMENTARY MATERIAL

Insights Imaging (2026) Zhang RN, Ke YQ, Cai HS, et al.

Supplementary Material 1: Psychological questionnaires

1) State-trait anxiety inventory (STAI)

The purpose of STAI is to measure via self-report the presence and severity of current symptoms of anxiety and a generalized propensity to be anxious. The measure consists of two subscales. The State Anxiety Scale (S-Anxiety) evaluates the current state of anxiety, while the Trait Anxiety Scale (T-Anxiety) evaluates relatively stable aspects of “anxiety proneness”. The scores of individual items are summed to calculate the subtest total scores. The score range for each subtest is 20–80, with higher scores indicating a higher level of anxiety.

State-Trait Anxiety Inventory (STAI-State score)[1]

	NOT AT ALL	SOMEWHAT	MODERATE SO	VERY MUCH SO
1. I feel calm	1	2	3	4
2. I feel secure	1	2	3	4
3. I am tense	1	2	3	4
4. I feel strained	1	2	3	4
5. I feel at ease	1	2	3	4
6. I feel upset	1	2	3	4
7. I am presently worrying over possible misfortunes	1	2	3	4
8. I feel satisfied	1	2	3	4
9. I feel frightened	1	2	3	4
10. I feel comfortable	1	2	3	4
11. I feel self-confident	1	2	3	4
12. I feel nervous	1	2	3	4
13. I am jittery	1	2	3	4
14. I feel indecisive	1	2	3	4
15. I am relaxed	1	2	3	4
16. I feel content	1	2	3	4

17. I am worried	1	2	3	4
18. I feel confused	1	2	3	4
19. I feel steady	1	2	3	4
20. I feel pleasant	1	2	3	4

State-Trait Anxiety Inventory (STAI-Trait score)

	ALMOST NEVER	SOMETIMES	OFTEN	ALMOST ALWAYS
21. I feel pleasant	1	2	3	4
22. I feel nervous and restless	1	2	3	4
23. I feel satisfied with myself	1	2	3	4
24. I wish I could be as happy as others seem to be	1	2	3	4
25. I feel like a failure	1	2	3	4
26. I feel rested	1	2	3	4
27. I am “calm, cool, and collected”	1	2	3	4
28. I feel that difficulties are piling up so that I cannot overcome them	1	2	3	4
29. I worry too much over something that really doesn’t matter	1	2	3	4
30. I am happy	1	2	3	4
31. I have disturbing thoughts	1	2	3	4
32. I lack self-confidence	1	2	3	4
33. I feel secure	1	2	3	4
34. I make decisions easily	1	2	3	4
35. I feel inadequate	1	2	3	4
36. I am content	1	2	3	4
37. Some unimportant thought runs through my mind and bothers me	1	2	3	4
38. I take disappointments so keenly that I can’t put them out of my mind	1	2	3	4
39. I am a steady person	1	2	3	4
40. I get in a state of tension or turmoil as I think over my recent concerns and interests	1	2	3	4

2) Perceived stress scale (PSS)

The purpose of PSS is to assess the extent to which respondents perceive their lives as unpredictable, uncontrollable, and overwhelming. The items in this scale inquire about your feelings and thoughts over the past month. The total scores can vary between 0 and 56, with higher scores indicating a higher level of perceived stress[2].

Perceived Stress Scale

	Never	Almost Never	Sometime	Fairly Often	Very Often
1. In the last month, how often have you been upset because of something that happened unexpectedly?	0	1	2	3	4
2. In the last month, how often have you felt that you were unable to control the important things in your life?	0	1	2	3	4
3. In the last month, how often have you felt nervous and “stressed”?	0	1	2	3	4
4. In the last month, how often have you dealt successfully with day to day problems and annoyances?	0	1	2	3	4
5. In the last month, how often have you felt that you were effectively coping with important changes that were occurring in your life?	0	1	2	3	4
6. In the last month, how often have you felt confident about your ability to handle your personal problems?	0	1	2	3	4
7. In the last month, how often have you felt that things were going your way?	0	1	2	3	4
8. In the last month, how often have you found that you could not cope with all the things that you had to do?	0	1	2	3	4
9. In the last month, how often have you been able to control irritations in your life?	0	1	2	3	4
10. In the last month, how often have you felt that you were on top of things?	0	1	2	3	4
11. In the last month, how often have you been angered because of things that were outside your control?	0	1	2	3	4
12. In the last month, how often have you found yourself thinking about things that you have to	0	1	2	3	4

accomplish?					
13. In the last month, how often have you been able to control the way you spend your time?	0	1	2	3	4
14. In the last month, how often have you felt difficulties were piling up so high that you could not overcome them?	0	1	2	3	4

Questionnaire completion guide: The questions in this scale ask you about your feelings and thoughts during the last month. In each case, you will be asked to indicate your response by placing an “X” over the circle representing HOW OFTEN you felt or thought a certain way. Although some of the questions are similar, there are differences between them and you should treat each one as a separate question. The best approach is to answer quickly. That is, don’t try to count up the number of times you felt a particular way, but rather indicate the alternative that seems like a reasonable estimate.

3) Beck Depression Inventory (BDI)

The BDI was derived from clinical observations about the attitudes and symptoms displayed frequently by depressed psychiatric patients and infrequently by nondepressed psychiatric patients (Beck et al., 1961). The clinical observations were consolidated systematically into 21 symptoms and attitudes which could be rated from 0 to 3 in terms of intensity. The items were chosen to assess the intensity of depression. BDI is scored by summing the ratings given to each of the 21 items: none or minimal depression is < 10; mild to moderate depression is 10-18; moderate to severe depression is 19-29; and severe depression is 30-63[3].

Beck's Depression Inventory

Beck's Depression Inventory		
1.		
	0	I do not feel sad.
	1	I feel sad
	2	I am sad all the time and I can't snap out of it.
	3	I am so sad and unhappy that I can't stand it.
2.		
	0	I am not particularly discouraged about the future.
	1	I feel discouraged about the future.

2 I feel I have nothing to look forward to.
3 I feel the future is hopeless and that things cannot improve.

3.

0 I do not feel like a failure.
1 I feel I have failed more than the average person.
2 As I look back on my life, all I can see is a lot of failures.
3 I feel I am a complete failure as a person.

4.

0 I get as much satisfaction out of things as I used to.
1 I don't enjoy things the way I used to.
2 I don't get real satisfaction out of anything anymore.
3 I am dissatisfied or bored with everything.

5.

0 I don't feel particularly guilty
1 I feel guilty a good part of the time.
2 I feel quite guilty most of the time.
3 I feel guilty all of the time.

6.

0 I don't feel I am being punished.
1 I feel I may be punished.
2 I expect to be punished.
3 I feel I am being punished.

7.

0 I don't feel disappointed in myself.
1 I am disappointed in myself.
2 I am disgusted with myself.
3 I hate myself.

8.

0 I don't feel I am any worse than anybody else.
1 I am critical of myself for my weaknesses or mistakes.
2 I blame myself all the time for my faults.

3 I blame myself for everything bad that happens.

9.

0 I don't have any thoughts of killing myself.
1 I have thoughts of killing myself, but I would not carry them out.
2 I would like to kill myself.
3 I would kill myself if I had the chance.

10.

0 I don't cry any more than usual.
1 I cry more now than I used to.
2 I cry all the time now.
3 I used to be able to cry, but now I can't cry even though I want to.

11.

0 I am no more irritated by things than I ever was.
1 I am slightly more irritated now than usual.
2 I am quite annoyed or irritated a good deal of the time.
3 I feel irritated all the time.

12.

0 I have not lost interest in other people.
1 I am less interested in other people than I used to be.
2 I have lost most of my interest in other people.
3 I have lost all of my interest in other people.

13.

0 I make decisions about as well as I ever could.
1 I put off making decisions more than I used to.
2 I have greater difficulty in making decisions more than I used to.
3 I can't make decisions at all anymore.

14.

0 I don't feel that I look any worse than I used to.
1 I am worried that I am looking old or unattractive.
2 I feel there are permanent changes in my appearance that make me look unattractive
3 I believe that I look ugly.

15.
0 I can work about as well as before.
1 It takes an extra effort to get started at doing something.
2 I have to push myself very hard to do anything.
3 I can't do any work at all.

16.
0 I can sleep as well as usual.
1 I don't sleep as well as I used to.
2 I wake up 1-2 hours earlier than usual and find it hard to get back to sleep.
3 I wake up several hours earlier than I used to and cannot get back to sleep.

17.
0 I don't get more tired than usual.
1 I get tired more easily than I used to.
2 I get tired from doing almost anything.
3 I am too tired to do anything.

18.
0 My appetite is no worse than usual.
1 My appetite is not as good as it used to be.
2 My appetite is much worse now.
3 I have no appetite at all anymore.

19.
0 I haven't lost much weight, if any, lately.
1 I have lost more than five pounds.
2 I have lost more than ten pounds.
3 I have lost more than fifteen pounds.

20.
0 I am no more worried about my health than usual.
1 I am worried about physical problems like aches, pains, upset stomach, or constipation.
2 I am very worried about physical problems and it's hard to think of much else.
3 I am so worried about my physical problems that I cannot think of anything else.

21.

0	I have not noticed any recent change in my interest in sex.
1	I am less interested in sex than I used to be.
2	I have almost no interest in sex.
3	I have lost interest in sex completely.

Supplementary Material 2: MR enterography protocol

CD patients underwent routine MRE. 1,500–2,000 mL of 2.5% mannitol solution was administered 1 hour before MRE. The buttocks were injected intramuscularly with 10 mg of raceanisodamine hydrochloride 10 minutes prior to MRE. MRE was performed using a 3.0 T MR system (MAGNETOM Prisma; Siemens Healthineers, Erlangen, Germany) with multi-channel phased-array body coils. T2-weighted imaging (T2WI), T2-weighted fat suppression sequence, and diffusion-weighted MRI (DWI) were performed. Pre-/post-enhancement T1-weighted imaging(T1WI) were acquired before and at 28 s (coronal), 70 s (coronal), 90 s (coronal), 3 min (axial), and 3.5 min (coronal) after the intravenous injection of 0.1mmol/kg Gadobutrol (Gadovist; Bayer Schering Pharma, Berlin, Germany) at a rate of 1.5-2.0 mL/s.

MRE sequences and parameters

Parameter	**T2WI HASTE**	**T2WI HASTE**	**T2WI HASTE fat suppression**	**DWI SE-EPI**	**Pre-/post-enhancement T1WI**
Orientation	2D axial	2D coronal	2D coronal	2D axial	3D coronal
Acquisition Matrix	380×267	450×422	450×422	380×283	450×394
Flip angle (°)	160	153	151	-	10.5
Slice thickness (mm)	4	4	4	4	2
Echo time (msec)	81	83	83	47	1.35, 2.58
Repetition time (msec)	1,000	800	800	5,400	4.28
Bandwidth (Hz/Pixel)	919	1,042	1,042	2,488	740, 740
Averages	1	1	1	1, 2, 2	1
Number of slices	45	27	27	45	88
Respiratory control	BH	BH	BH	Free breathing	BH

b factors (sec/mm^2)	-	-	-	50,400,800	-
Acquisition time (sec)	63	24	24s	105	23

T2WI, T2-weighted imaging; HASTE, half-Fourier acquisition single-shot turbo spin echo; DWI, diffusion-weighted MRI; MRI, magnetic resonance imaging; SE-EPI, spin-echo echo-planar imaging; 2D, two-dimension; 3D, three-dimension; BH, breath hold.

Supplementary Material 3: Assessment of intestinal inflammation severity of patients with CD

The MRE images, targeting the most severely diseased intestine, were evaluated by three senior abdominal radiologists (each with >10 years of MRE experience). To ensure a blinded interpretation, the following steps were taken: First, an independent research coordinator removed all patient-identifying information and created a de-identified image dataset for this study. Second, the three radiologists independently assessed this dataset in a randomized order on separate workstations, without access to the clinical Picture Archiving and Communication System or electronic medical records. They were explicitly instructed not to consult clinical teams during the review. Finally, a consensus meeting was held to reconcile any discrepant findings, during which the radiologists remained blinded to all non-imaging data.

1) Magnetic resonance enterography global score (MEGS)

When using MEGS to assess the severity of intestinal inflammation, the bowel was divided into nine segments (including jejunum, ileum, terminal ileum, caecum, ascending colon, transverse colon, descending colon, sigmoid colon, and rectum). The scoring of each segment was independently conducted by three experienced radiologists in consensus, who were blinded to the endoscopic and neural findings, according to the following table. Individual segmental scores were summed and then 5 points added if lymph nodes ≥1 cm (short axis diameter), comb sign, fistulae (entero-enteric, entero-cutaneous or entero-vesical) or abscesses were present. The final summed score was termed MEGS[4].

Magnetic resonance enterography global score[4]

Score	0	1	2	3
Mural thickness*<br>small bowel	<3 mm	>3–5 mm	>5–7 mm	>7 mm
Mural T2 signal**	Equivalent to normal	Minor increase in signal:	Moderate increase in signal:	Marked increase in signal:

	bowel wall	bowel wall appears dark grey on fat-saturated images	bowel wall appears light grey on fat-saturated images	bowel wall contains areas of white high signal approaching that of luminal content
Peri-mural T2 signal (mesenteric oedema)	Equivalent to normal mesentery	Increase in mesenteric signal but no fluid	Small fluid rim (≤2 mm)	Larger fluid rim (>2 mm)
T1 Enhancement***	Equivalent to normal bowel wall	Minor enhancement: bowel wall signal greater than normal small bowel but significantly less than nearby vascular structures	Moderate enhancement: bowel wall signal increased but somewhat less than nearby vascular structures	Marked enhancement: bowel wall signal approaches that of nearby vascular structures
Mural enhancement pattern	N/A or homogeneous	Mucosal	Layered	
Haustral loss (colon only)	None	<1/3 segment	1/3 to 2/3 segment	>2/3 segment
**Multiplication factor per segment**				
Length of disease segment		0–5 cm × 1	5–15 cm × 1.5	>15 cm × 2
**Additional score for extramural features**				
**Score**	**0**	**5**		
Lymph nodes (≥1 cm measured in shortest diameter)	Absent	Present		
Comb sign (linear densities on the mesenteric side of affected bowel segments)	Absent	Present		
Abscess	Absent	Present		
Fistulae	Absent	Present		

*Measured using electronic callipers; **compared with normal small bowel; ***compared with nearest vessel

2) Magnetic Resonance Index of Activity (MaRIA)

When using MaRIA to assess the severity of intestinal inflammation, the bowel was divided into six segments (including distal ileum, ascending, transverse, descending, sigmoid colon and rectum). The MaRIA score for each segment was calculated independently by three experienced radiologist who were blinded to the endoscopic, MEGS and neural findings using the following formula: MaRIA (segment) = 1.5×wall thickness (mm)+0.02×relative contrast enhancement+5×oedema+10×ulceration, where mural oedema was defined as hyperintesity on T2-wedged sequences of the wall relative to the signal of the psoas muscle, and mucosal ulceration was defined as deep depressions in the mucosal surface[5]. The calculation of relative contrast enhancement was described in detail in the previous study[5]. The highest MaRIA among the inflamed intestines were selected to characterize the severity of intestinal inflammation in CD patients.

Supplementary Material 4: Blood neurotransmitter assessment

The procedure began with the thawing of samples on an ice bath. Samples included serum from 105 patients with CD and 30 age- and sex-matched healthy controls. Subsequently, 25 μl of each plasma sample was transferred to a 96-well plate. The automation process was carried out using the Biomek 4000 workstation from Beckman Coulter, Inc., following a preprogrammed protocol. In summary, 100 μl of ice-cold methanol containing partial internal standards was automatically added to each sample, followed by vigorous vortexing for 5 minutes. The plate underwent centrifugation at 4000 g for 30 minutes using the Allegra X-15R centrifuge from Beckman Coulter, Inc., based in Indianapolis, USA. Following centrifugation, 30 μl of the supernatant was carefully transferred to a clean 96-well plate, and 20 μl of freshly prepared derivative reagents were

added to each well. The plate was sealed, and derivatization occurred at 30 ◦C for 60 minutes. Post-derivatization, 350 μl of ice-cold 50% methanol solution was added to dilute the sample. Subsequently, the plate was stored at -20 ◦C for 20 minutes, followed by a centrifugation step at 4000 g and 4 ◦C for 30 minutes. Quantitation of the metabolite was performed using an ultraperformance liquid chromatography coupled to tandem mass spectrometry (UPLC-MS/MS) system (ACQUITY UPLC-Xevo TQ-S, Waters Corp., Milford, USA). Due to the rapid turnover of many intracellular metabolites, immediate metabolism quenching was essential. Extraction solvents were pre-chilled in a -20 ℃ freezer overnight and added to the samples immediately upon thawing. An ice-salt bath was employed to maintain a low temperature during sample preparation, minimizing degradation risks. All prepared samples were analyzed within 48 hours after extraction and derivatization. Stringent quality control/assurance (QC/QA) procedures were implemented, ensuring consistent high-quality analytical results. These procedures encompassed meticulous control at every stage, from sample receipt in the laboratory to the delivery of the final results. The objective of the Quality Control/Quality Assurance (QC/QA) measures was to ensure the generation of reliable data for the biomarker discovery study. This involved the incorporation of three distinct types of quality control samples—namely, test mixtures, internal standards, and pooled biological samples—within the metabolomics platform. To further enhance the robustness of the analytical process, conditioning samples, and solvent blank samples were also integrated to optimize instrument performance. For meticulous sample tracking and management, each sample underwent accession to the Metabo-Profile Laboratory Information Management System (LIMS). Subsequently, the LIMS system assigned a unique identifier to each sample, facilitating the comprehensive tracking of all aspects, including sample handling, tasks, results, and other relevant conditions. The implementation of barcoding for both samples and

aliquots further streamlined the process, enhancing efficiency and accuracy in sample identification and analysis under the purview of the LIMS system.

The acquired GC–TOF/MS data were processed using a XploreMET v3.0 system (Metabo-Profile, Shanghai, China). Partial least squares projection to latent structures and discriminant analysis (PLS-DA) were employed for data analysis. Principal component analysis (PCA) and partial least square discriminant analysis (PLS-DA) were conducted on both positive and negative models after log transformation and pareto scaling. The variable importance in the projection value of each variable in the PLS-DA model was calculated to indicate its contribution to the classification task. Metabolites with VIP values > 1 were assessed for significance using Student's t-test at the univariate level, and the results were adjusted for multiple testing using the Benjamini–Hochberg procedure with a critical false discovery rate (FDR) set at 0.05.

References

1 Julian LJ. Measures of anxiety: State-Trait Anxiety Inventory (STAI), Beck Anxiety Inventory (BAI), and Hospital Anxiety and Depression Scale-Anxiety (HADS-A). *Arthritis Care Res (Hoboken)* 2011;63 Suppl 11(0 11):S467-S72.

2 Cohen S, Kamarck T, Mermelstein R. A global measure of perceived stress. *J Health Soc Behav* 1983;24(4):385-96.

3 Beck AT, Steer RA, Brown GK. Beck Depression Inventory (2nd ed.). 1996.

4 Makanyanga JC, Pendsé D, Dikaios N, et al. Evaluation of Crohn's disease activity: initial validation of a magnetic resonance enterography global score (MEGS) against faecal calprotectin. *Eur Radiol* 2014;24(2):277-87. Epub 20130912.

5 Rimola J, Rodriguez S, García-Bosch O, et al. Magnetic resonance for assessment of disease activity and severity in ileocolonic Crohn's disease. *Gut* 2009;58(8):1113-20.
